# Where Lies the Risk? An Ecological Approach to Understanding Child Mental Health Risk and Vulnerabilities in Sub-Saharan Africa

**DOI:** 10.1155/2014/698348

**Published:** 2014-04-16

**Authors:** Olayinka Atilola

**Affiliations:** Department of Behavioural Medicine, Lagos State University College of Medicine, Ikeja, Lagos 10001, Nigeria

## Abstract

Efforts at improving child-health and development initiatives in sub-Saharan Africa had focused on the physical health of children due to the neglect of child and adolescent mental health (CAMH) policy initiatives. A thorough and broad-based understanding of the prevalent child mental-health risk and vulnerability factors is needed to successfully articulate CAMH policies. In this discourse, we present a narrative on the child mental-health risk and vulnerability factors in sub-Saharan Africa. Through an ecological point of view, we identified widespread family poverty, poor availability and uptake of childcare resources, inadequate community and institutional childcare systems, and inadequate framework for social protection for vulnerable children as among the risk and vulnerability factors for CAMH in the region. Others are poor workplace policy/practice that does not support work-family life balance, poor legislative framework for child protection, and some harmful traditional practices. We conclude that an ecological approach shows that child mental-health risks are diverse and cut across different layers of the care environment. The approach also provides a broad and holistic template from which appropriate CAMH policy direction in sub-Saharan Africa can be understood.

## 1. Introduction

The last century witnessed considerable changes in the nature and pattern of child and adolescent health problems, significant among which is the unequivocal recognition of mental disorders as a source of childhood morbidity [1]. This recognition is not unconnected with emerging evidence from multinational epidemiological surveys that mental and behavioural problems among children and adolescents are common and a worldwide phenomenon [2]. Childhood mental disorders are also associated with significant distress to the child and a major burden to the society [3–5]. Though there is yet to be any conclusive evidence that childhood mental disorders are relatively more prevalent in low-resource countries, there are indicators to suggest that the global burden of childhood mental health problems is likely to be concentrated in low- and middle-income (LAMI) countries. In the first instance, 85% of the world's child and adolescent population live in the LAMI countries [6]. Secondly, the LAMI regions of world have poorer child-related social indicators [7], a situation which can increase the risk of childhood mental health problems [8].

Among the LAMI regions of the world, children living in sub-Saharan Africa in particular face a life of poverty, poor nutrition, and social problems which can impair their early development as well as their mental health and wellbeing [9]. Currently available epidemiological evidence shows a prevalence rate as high as 20% for childhood mental health problems in sub-Saharan Africa [10]. Perhaps inevitably, efforts at improving on the poor state of child-health and development initiatives in sub-Saharan Africa had focused on improving the physical health of children. This focus has however been on the neglect of child and adolescent mental health (CAMH) policy development as a child-health and development initiative in the region. Part of the problem is the poor understanding of CAMH issues and awareness of the consequences of child mental health problems among policy makers. As a result, CAMH service and policy development have remained low in the rungs of public-health thrust in sub-Saharan Africa [11–13]. Child and adolescent mental health policies are critical component of the healthcare delivery system of nations [12], without which preventive and restorative child mental health services cannot be hatched on an integrated platform which can ensure success. Children will therefore suffer from preventable mental health problems, while those in need of curative services may not get it. Untreated mental health problems among children constitute major social and economic burden on families and the society at large [14, 15].

Articulating a CAMH policy for any region requires a thorough and broad-based understanding of the risk and vulnerability factors for child mental health problems in such region. In fact, in other climes, such effort has been identified as a unique step towards charting a course for CAMH policy development [16–18]. Researchers working in LAMI regions have also adopted a risk and vulnerability approach to set directions for CAMH service and policy development in recent times [19]. However, there had not been any concise documentation of child mental health risk and vulnerability points in sub-Saharan Africa. This is a critical omission for a region with almost half a billion child population [20], majority of whom are also living in sundry difficult social circumstances [7]. Therefore, with a view to emphasize the appropriate target points for CAMH policy development in sub-Saharan Africa, we present a narrative on the child mental health risk and vulnerability points in the region. To ensure capturing the ramifications of risk and vulnerability points as much as possible, we adopt the Ecological Model of Childhood [21] as the descriptive framework.

## 2. Ecological Model of Child and Adolescent Mental Health Risk and Vulnerability Factors in Sub-Saharan Africa 

Bronfenbrenner's Ecological Model of Childhood [21] compartmentalized the operating milieu and the care environment of the child into 5 hierarchal but interconnected layers. These include the microsystem, mesosystem, exosystem, macrosystem, and time bound chronosystems in that order of hierarchy. The risk and vulnerability points for child mental health in the different components of the ecological care environment of the child are hereby examined.

## 3. Risk and Vulnerabilities within the Micro- and Mesosystem

In this section, peculiar risk and vulnerability points for CAMH in sub-Saharan Africa are examined starting from the family unit, being the most critical component of the ecological care environment of children [22]. Other components of the microsystem examined include the school, other childcare facilities, and the neighbourhoods where children grow up. The mesosystem of the care environment of a child is formed mainly by the quality of interaction between the agents within the microsystem. These interactions include the degree of uptake and utilisation of available childcare resources.

### 3.1. Poorly Resourced Families as a Risk and Vulnerability Point

Among the key components of the microsystem of the care environment of a child is the family unit. In fact, the intricate relationship between the child and the family unit shapes all aspects of children's physical and mental development [22]. The family unit has also been described as a critical component of the care environment of children which ensures they receive the appropriate nurturance for good social and health outcomes [23]. Therefore, the presence of an appropriate, resourceful, and stable family is a key resilience factor for the mental health and wellbeing of a child. The ability of the family unit to provide optimal care for children however depends on the level of resources, including financial and social, that are available to them. Unfortunately, there are indications that a large proportion of children in sub-Saharan Africa are being nurtured under caregiving or family arrangements that may not guarantee optimal social and mental development.

To start with, about 55 million of children in sub-Saharan Africa are orphans who live in different forms of alternative care [24]. Going by the current child population of about 430 million in the region [20], this translates to about 13% of all children in the region. The sheer burden of orphans and other out-of-care children in the region is believed to have overstretched the traditional extended-family system in the region, giving rise to different forms of alternative care arrangements including child-headed households [25, 26]. Although the exact estimate is not available, many of the orphaned children in sub-Saharan Africa live in child-headed households [27, 28]. Consistent with studies from the developed countries which show that children who grew up in alternative care settings experience poorer mental health [29, 30], a study conducted in Zambia showed that children being raised in child-headed households had poorer indicators of health (including mental health) compared with their peers growing up in adult-headed households [31]. Besides the risk of being brought up in alternative care arrangements, orphans are ordinarily at risk of cumulative social disruptions and traumatic events [32] which may put them at higher risk of mental health problems. It is therefore not surprising that cross-sectional and longitudinal studies from different parts of sub-Saharan Africa have found higher prevalence rates of mental disorders among orphaned children compared with nonorphaned controls [32–34].

Moreover, a significant proportion of children in sub-Saharan Africa are born into “families” with limited childcare resources. For instance, up to 123 out of 1000 children in the region are born to children below 18 years [7]. Many of the children so born also grow up in alternative care arrangements with their attendant mental health risks. Besides this, the quality of care that can be provided by a child-mother is limited so is the likelihood of full utilisation of childcare resources. Therefore, being born to a child and being a child-mother both carry mental health risks. In a review of child mental health problems in sub-Saharan Africa, lower maternal age was an independent predictor [10].

Equally relevant is the fact that the well-known strong inclination towards a stable family life in sub-Saharan Africa is already on a fast decline [35], giving rise to an emerging generation of single-parent households in the region [36, 37]. A recent survey in South Africa, for example, found that up to 40% of children are living in single-parent households [38]. Though there are cultural differences in what constitutes the ideal family, the chances of optimal mental health and wellbeing of children are most guaranteed in a cohesive dual-parent setting [39]. Large-scale studies from the developed countries have found a higher prevalence of mental health problems among children in single-parent families [40]. Similar large-scale studies are yet to be conducted in sub-Saharan Africa but small-scale studies have established links between family-life deficits and adverse social and mental health outcomes for children [41, 42].

Furthermore, about 50% of families and household in sub-Saharan Africa live on less than USD 1.25 per day [7]. Severe limitation of financial resources can impair the ability of families to meet the social and emotional needs of children [43]. Poverty can also initiate a spiral of events that can threaten the stability of the family unit [44, 45] and as such can put the social and mental wellbeing of children at risk. A good illustration can be found in the sundry reports from many parts of sub-Saharan Africa that parental poverty is the key factor responsible for children dropping out of school, living on the street, being forced into early marriage, engaging in child labour, and living in sundry other difficult circumstances [46–49]. Therefore, by fostering family instability or putting children in precarious situations, family poverty is one of the factors within the ecological care environment that is capable of increasing the mental health risk of children. In a recent review of all the available epidemiological data on mental health problems among children in sub-Saharan Africa, most of the studies reviewed found an association between childhood psychopathology and poorer parental socioeconomic status [10]. Besides this, the highest burden of mental health problems among children in sub-Saharan Africa has been found among children living in sundry poverty-driven difficult circumstances. For instance while the prevalence of mental health problems among the general population of children in sub-Saharan Africa currently hovers around 10–30% [10], studies conducted in the region have found as high as 50–90% rate of mental health problems among children living in poverty-driven difficult circumstances like street children [50], child labourers [51], and children living on social welfare or in juvenile justice custody [52, 53].

Besides material resources, educational resources of care givers are also important in determining the quality of childcare, including the uptake of available resources for childcare. General literacy can improve awareness and uptake of newer childcare insights and practices. The adult literacy rate which is as high as 99% in some developed countries averages about 63% in sub-Saharan Africa [54, 55]. In fact, adult literacy is as low as 45% among women in the region [54, 55]. The figures for women can even be as low as 10% or less in some sub-Saharan Africa countries like Niger and Burkina Faso [55]. Without prejudice to the rich indigenous knowledge for childcare in sub-Saharan Africa which may have nothing to do with formal schooling [56], education—in terms of literacy—often serves as a proxy for knowledge and information as well as cognitive skills and societal values [57]. All these can influence good childcare choices [58]. Therefore, parental illiteracy is capable of limiting the full utilisation of available childcare resources and as such limiting the mental health reserve of their children.

### 3.2. Poor Availability and Uptake of Childcare Resources as Risk and Vulnerability Point

There is substantial evidence that the availability and uptake of Early Childhood Care and Development (ECCD) resources are key determinant of social and mental health outcomes for children [8]. In this context and thereafter, ECCD is understood as resources for health promotion, optimal nutrition, psychosocial, and cognitive stimulation, as well as other supports to strengthen the care environment of children [59]. Common ECCD resources usually include health and educational resources, maternal health services, and child nutritional support among others. Though significant effort is being made, a complex interplay of social, economic, structural, and cultural factors continues to limit availability and utilisation of ECCD resources in sub-Saharan Africa [60]. Lack or poor utilisation of ECCD resources can impair the resilience of children against physical and mental health shocks [8]. Therefore, from mental health perspectives, lack of or poor utilisation of ECCD resources in sub-Saharan Africa suggests that a large proportion of children in the region may be disadvantaged in terms of baseline resilience or mental health reserves.

Specifically, only about half of mothers in sub-Saharan Africa utilise or have access to antenatal care services or skilled birth attendance on the average [7]. The rate of antenatal-care access and utilisation is as low as 20% in some countries like Niger and Chad, while skilled birth attendance is lower than 40% in Nigeria, Chad, Ethiopia, and Niger [7]. Studies from Kenya [61] through Sudan [62] to Nigeria [63] have established a link between poor antenatal care or lack of skilled birth attendance and poor obstetric outcomes. Obstetrics complications arising from poor maternal care in sub-Saharan Africa are the key risk factor for perinatal asphyxia [64], which is very common in the region [65]. Some studies from sub-Saharan Africa had found an association between perinatal asphyxia and childhood mental disorders [66].

Similarly, up to 40% of children below 5 years in the sub-Saharan Africa are malnourished, while up to 20% do not receive complete immunisation by respective national guidelines [7]. Nutritional deficiencies and lack of full immunisation put children at a risk of childhood infectious diseases like measles, poliomyelitis, meningitis, and others which can lay a foundation for mental health problems in later childhood. On a different note, high-quality early education provided in a highly stimulating environment is expected to come with an early sense of self-esteem and confidence and a repertoire of problem-solving skills which are critical protective factors for mental health [67]. Therefore, quality early child education for instance is a fundamental mental health resilience factor. Unfortunately, access to and utilisation of early-childhood educational resources are still poor in sub-Saharan Africa. The preprimary school Gross Enrolment Rate (GER) in sub-Saharan Africa averages about 18% while the primary school Net Enrolment Ratio (NER) averages 66% [7]. Though at 86%, the rate of survival to the last grade in primary school is fair, an average secondary school NER of about 27% in the region [7] erodes this apparent gain.

Besides this, infrastructure for early education in sub-Saharan Africa is poor, and the average pupil-teacher ratio of 45 : 1 in the region is the highest in all the regions of the world [68]. In addition, a survey of teacher motivation and satisfaction also found that majority of elementary school teachers in sub-Saharan Africa are dissatisfied with their jobs and are poorly motivated [69]. This situation has continued to threaten the proper acquisition of early childhood foundational education and social-skill development in many parts of sub-Saharan Africa [69]. Poor pupil-teacher interaction, which can be envisaged in a situation of high pupil-teacher ratio and poor teacher motivation, reduces the chances of the child benefitting fully from the mental health benefits of school participation. It can also reduce the chances of early detection of emotional and behavioural problems [70].

Likewise, in the event that childhood mental health problem arises, availability of mental healthcare facilities is crucial. This is because untreated mental health problems among children hardly just go away; rather they are associated with social and functional impairments [4]. Therefore, availability of restorative CAMH services is a good child mental health resource. Unfortunately, resources for restorative mental health service are very limited in sub-Saharan Africa, in terms of both human and material resources [13]. Many countries in the region have an acute shortage of CAMH facilities and an abysmally low number of trained CAMH practitioners, sometimes in the ratio of 1 : 10 million child population [71]. To compound the problem, some recent studies have reported a poor utilisation of the few available mental health services for children [50].

### 3.3. Risk and Vulnerabilities in the Living Neighbourhood

The neighbourhood in which children grow is also a critical component of the microsystem. The neighbourhood is part of the sources of stimulation, care, and nurturance, as well as an embodiment of values and behavioural models for children. Therefore, the neighbourhoods within which children grow up contribute significantly to children's mental health and wellbeing. Poverty, social inequalities, and poor rural development have created a situation of exodus of people to the urban areas of sub-Saharan Africa. As a result, urbanized population in sub-Saharan Africa has been on the rise. Urbanized population is as high as 50% in Nigeria and Cote d'Ivoire and over 60% in South Africa, Congo, and Cameroon [7]. In fact, with a current population of at least 10 million, Lagos (Nigeria) has acquired the status of a megacity while Nairobi (Kenya) with an annual population growth rate of 13% may soon join the league [72]. Urbanisation, especially very rapid ones, poses a lot of mental health challenges for vulnerable group of individuals, including children, in LAMI countries [73].

Many of the rapidly urbanising cities in sub-Saharan Africa lack the facilities to cater for the large influx of persons, as such, creating a situation of urban slums in many parts of the region [74]. More than 70% of urban dwellers in sub-Saharan Africa live in slums [75], expectedly with their children. This proportion is the highest for any region in the world [75]. Urban slums in low-income regions like sub-Saharan Africa are associated with lack of basic amenities, poor housing, unsafe neighbourhoods, and social exclusion [76]. These social circumstances constitute mental health risk for children. Reports from other parts of the world had found that children living in slums had poorer mental health [77, 78]. A recent study conducted among children and youth in the slums of Kampala (Uganda) found a prevalence of suicidal ideation that is very much higher than the national average [79].

## 4. Risk and Vulnerabilities in the Exosystem

The key components of the exosystem examined here include the parental workplace, community childcare facilities like social welfare and juvenile justice systems, and social-protection schemes for children.

### 4.1. Poor Workplace Policies as a Vulnerability and Risk Factor

Sub-Saharan Africa is among the worst-hit regions by the shock waves of the global economic crisis [80]. The crippling effect of the global economic crisis on the economy is further complicated by the preexisting low infrastructural capacity for industrialization and job creation in the region. As a result, there is an intensive scampering for jobs in the region which creates a situation for exploitative and unhealthy working conditions [81]. Furthermore, the labour market in sub-Saharan Africa is characterized by high level of precarious forms of employment like casualised labour [82, 83] and forced labour [84]. A lot of women in sub-Saharan Africa had had to augment family income by participating in the informal and formal labour market [85]. This often comes at some cost to childcare, as women are traditionally the primary caregiver for children in the region [86].

Workplace policies in sub-Saharan Africa have not fully embraced initiatives on family-work life balance as stipulated by International Labor Organisation (ILO) Conventions [87]. For instance, none of the countries in sub-Saharan Africa make provision for parental leave which allows both parents to take paid leave to care for children [87]. Also, only a few countries in the region provide up to the recommended 14 weeks maternity leave. In fact, it is as low as 60 days in countries like Eritrea, Guinea-Bissau, Kenya, Malawi, Mozambique, and Uganda. There is also a deficient—if not nonexistent—framework for extending paid maternity leave to workers in the informal sector in sub-Saharan Africa, which is the main employer of women in the region [88]. In the context of Bronfenbrenner's ecological model of childhood, the lack of opportunity for sharing quality time with children and the instability and unpredictability of family life that is created by precarious employment have been described as the most destructive force to child physical and mental wellbeing globally [89]. Therefore, the precarious nature of* work* and* working* in sub-Saharan Africa can be viewed as a significant potential source of risk for mental health and wellbeing of children in the region.

### 4.2. Lack of Adequate Community and Institutional Childcare Systems as a Risk Factor

Among the repercussions of the ongoing global economic crisis is increased incidence of child maltreatment/neglect, delinquency, and youth crime [90, 91]. In the last decade, the proportion of children coming in contact with the social welfare and juvenile justice systems in the sub-Saharan Africa as a result of neglect or delinquency had been on the rise [52, 92, 93]. The availability of a well-organized juvenile justice and social welfare system offers a good opportunity for many of these children and adolescents to receive the necessary care, guidance, mentorship, reformation, and mental health services needed for their rehabilitation and reintegration back into the society.

Unfortunately, despite a high need for juvenile justice and child social-welfare services in many parts of sub-Saharan Africa, these systems are still poorly developed in the region [42, 94, 95]. The care of children in contact with social welfare and juvenile justice institution in the region is still hinged largely on punitive incarceration or institutional seclusion without much framework for true reformation or addressing their social and mental health needs [42, 96]. The implication is that there currently exist a limited capacity and framework in the sub-Saharan Africa region for assisting children and adolescents who may be out-of-care as a result of maltreatment/neglect or in need of reformation as a result of delinquency/youth crime to regain their footing for continued mental health and wellbeing.

### 4.3. Lack of Adequate Social Protection for Vulnerable Children as a Risk Factor

In a care environment associated with deprivation, social insecurity, and social inequalities, social protection schemes for families and children offer a unique and effective policy framework to mitigate the damaging effects on the mental health and wellbeing of children and their families. This fact is recognized by the 2005 report of the Commission for Africa [97]. Social protection schemes can mitigate the social and mental health risks of poverty and inequalities on children and their families. In spite of the modest expansion in Eastern and Southern regions of Africa in recent times [98], available data unfortunately suggests that social protection and social-welfare schemes in sub-Saharan Africa are still largely rudimentary, noncomprehensive, restrictive, or at experimental stages [99–101]. In addition, the social protection schemes in the region are still bedeviled by weak institutional framework for impactful implementation [102]. This scenario has the potential of limiting the capacity of households in sub-Saharan Africa to break the poverty cycle, provide qualitative care for children, and ensure the mental wellbeing of children.

## 5. Risk and Vulnerability Points in the Macrosystem

Though it is the most distant component of the care ecosystem of a child in terms of the proximity of impact, the components of the macrosystem affect the wellbeing of children through its influence on the components of the other layers of the care environment. The components of the macrosystem include cultural practices relating to children, legislative framework for the protection of children, and globalisation and its effect on children, among other things.

### 5.1. Cultural Practices in Childcare as a Risk Factor

Many of the cultures in sub-Saharan Africa place a lot of premium on childcare and have a lot of healthy practices that can enhance the mental health of children [103, 104]. In fact, some of the peculiar parenting styles in sub-Saharan Africa which promote sense of duty and responsibility, as well as subservience to culturally recognized authorities, have been speculated to promote mental wellbeing of children [103]. There are however a lot of potentially harmful traditional practices in the region that can affect the mental health of children. For instance, widespread cultural and religious beliefs in many parts of sub-Saharan Africa have entrenched corporal punishment and other forms of abusive and potentially abusive methods of child control [104]. Though the psychological effects of corporal punishment among children in sub-Saharan Africa are yet to be systematically studied [104, 105], studies from other parts of the world show that corporal punishments have deleterious effects on mental health of children [106, 107]. Similarly, linked to deep-seated and ancestrally entrenched cultural beliefs, close to 3 million girls are estimated to undergo female genital mutilation annually in sub-Saharan Africa [108]. Besides the sundry physical health implications of this traditional practice [109], harmful effects on mental health of girls and women have been documented [110].

In addition, several studies have shown that women and children are disinherited through various traditional and cultural norms in sub-Saharan African communities [111–113]. Life-course analyses have however established the importance of asset transfers in intergenerational wellbeing of persons [114, 115]. Intergenerational studies of poverty transmission in sub-Saharan Africa have found disinheritance of women and children as a perpetuating factor, with attendant adverse implications for the mental health and wellbeing of children and women [116]. Furthermore, driven by practice that is linked with strongly held cultural and religious values, sub-Saharan Africa also has one of the highest rates of child marriages in the world [117]. Child marriage can interfere with proxies of mental health like education and free socialisation and can put the child-wife in situations that increase mental health risks [118]. Other traditional practices in sub-Saharan Africa that can impact negatively on the mental health and wellbeing of children include, but not limited to, taboos against consumption of some nutritious foods by pregnant women and hierarchical or gender based distribution of food [105]. Cultural and religious beliefs are also known to contribute to delays in seeking medical care for childhood mental health problems in the region [50].

### 5.2. Poor Legislative Framework for Child Protection as a Risk Factor

The legislative framework for the protection of the wellbeing of children in a country is a component of the macrosystem of the care environment of a child. Besides a few regional charters for the protection of the rights of children, the provisions of the United Nation's Convention on the Rights of Children [119] are the framework for achieving this objective globally. The Convention on the Rights of Children (CRC) seeks to promote the physical, mental, and social wellbeing of children by conferring certain rights on children which state parties are expected to strive to protect. This legislative framework is expected to compel state parties to invest in the physical, mental, and social wellbeing of children by creating the necessary socioeconomic and legislative milieu for the protection and assurance of the rights of the child. With the exception of Somalia, all sub-Saharan African countries signed, ratified, and domesticated the CRC. However, many of these countries have not been able to fully guarantee these rights due to a combination of lack of adequate local legislative framework for operationalisation, poor political will, sociocultural barriers, and poor socioeconomic realities [120, 121]. These barriers to full implementation of child's rights continue to put children in the region at significant mental health risks. This is because most of the factors like child abuse and neglect, child labor, and lack of access to ECCD, which put children at mental health risks, are preventable within the framework of child rights.

### 5.3. Globalisation and Its Effect on Childhood as a Risk Factor

The macrosystem level of the care environment of children in resource-poor regions of the world like sub-Saharan Africa is highly susceptible to disruptions arising from the globalizing influence of the world's dominant socialization. It is a well-known fact that for a long time, the spirit of “*ubuntu*” (one for all and all for one) has provided a strong bonding and a source of social, emotional, and material security for children and families during crisis periods in sub-Saharan African communities [122]. However, globalisation has engendered the emergence of elitist nuclear family system in sub-Saharan Africa and has created an increasing sense of individualism that undermines the spirit of* ubuntu* [123, 124]. In addition, economic globalisation has also widened the inequality gaps in many poor-resourced regions like sub-Saharan Africa [125] to the detriment of the mental health and wellbeing of children.

Despite the fact that cultural value and beliefs are known to influence the idioms of expression as well as manifestation of mental disorders [126], globalisation of mental healthcare has created a situation whereby childhood mental disorders in a non-Western regions like sub-Saharan Africa are increasingly being conceptualized from Western perspectives [127]. This has a potential of undermining an effective and culturally nuanced diagnosis and management of childhood mental health problems in the region. Also, some globalization-driven economic policy advice like the structural adjustment programme, deregulation and privatization policies, and currency devaluation initiatives has created disastrous economic blunders [128] which have affected the capacities of many sub-Saharan African countries to meet obligations on welfare and wellbeing of families and children [129].

## 6. Implications for Child and Adolescent Mental Health Policy Development in Sub-Saharan Africa

Though the planned focus of this paper is to be an expository on the risk and vulnerability points for child mental health for future development of CAMH policies, we will however make some general notes on the implications of the current discourse for CAMH policy development in the region. Suffice to say however that the ecological approach to CAMH risk and vulnerability points adopted in this discourse provides a broad and holistic template from which an angle to CAMH policy direction in the region can be understood. It also shows that approaches to CAMH policy development in the region must be cognizant of the fact that child mental health risks are diverse and cuts across different layers of the care environment. In line with recent recommendations for developing countries [130, 131] therefore, CAMH policy formulation in sub-Saharan Africa needs to have a multisectoral and multidisciplinary focus. This should include assigned roles for sectors including health, education, social welfare, juvenile justice, and legislative authorities. The Labor Office, environmental agencies, and community-based organizations among others will also have a role to play.

For the constraint of space we have dedicated another paper to charting a tentative course for CAMH policy in sub-Saharan Africa based on the risk and vulnerability points and using the Bronfenbrenner's ecological model of childhood as the operational framework [132]. Strategies targeted towards the micro-/mesosystem that have been recommended include but are not limited to strengthening the care capacity of the family unit through child-sensitive social-protection schemes extended to the most vulnerable families and households [132]. Significant emphasis should also be placed on providing for and removing barriers to accessing ECCD resources. There is ample evidence from sub-Saharan Africa that bridging the ECCD gap has a potential of reducing social inequalities, promoting the mental health and wellbeing of children, and breaking the intergenerational cycle of poverty [60]. Strategies to address risk and vulnerabilities in the exo- and mesosystems of the care environment of children should incorporate efforts and legislations to promote child-friendly workplace policies, strengthening the child social welfare and juvenile justice systems, ensuring further development of legislative frameworks to protect child rights, as well as community engagements on harmful traditional practices [132].

## 7. Conclusion

We have presented a narrative on the risk and vulnerability points for child mental health in sub-Saharan Africa, using an ecological approach. The risk and vulnerability points are broad, and cuts across many layers of the child's care environment. This underscores the need for CAMH policy development in the region to be multisectoral and multidisciplinary in design. While the social circumstance of children may differ among countries of sub-Saharan Africa, the general principles of this discourse will come in handy as each country evolves a CAMH policy.
